# Multicolour imaging signatures to diagnose concurrent macular telangiectasia type 2 and macular branch retinal vein occlusion

**DOI:** 10.3205/oc000125

**Published:** 2019-11-20

**Authors:** Ike Schouten, Sugandha Goel, Kumar Saurabh, Rupak Roy

**Affiliations:** 1 Department of Vitreo Retina, Aditya Birla Sankara Nethralaya, Kolkata, India

**Keywords:** multicolour imaging, blue reflectance imaging, macular telangiectasia type II, branch retinal vein occlusion

## Abstract

Concurrent macular telangiectasia type 2 (MacTel) and branch retinal vein occlusion (BRVO) have not been described before and can pose a diagnostic dilemma. The current report highlights the role of multicolour (MC) imaging and blue reflectance (BR) imaging in diagnosing this multi-pathology. A 60-year-old male presented with diminution of vision in the right eye. BR imaging showed parafoveal hyperreflectance, characteristic of MacTel, and hyperreflective parafoveal retinal vessels indicating vascular changes in RVO. MC imaging, particularly the BR component is a promising tool to aide in the diagnosis of concurrent MacTel and BRVO, guiding treatment decisions and prognosticating treatment outcomes.

## Introduction

Macular telangiectasia type 2 (MacTel) is a disorder characterised by macular neurodegeneration with more prominent associated vascular changes [1], [2]. MacTel with concurrent central serous chorioretinopathy has been described [3], [4]. But co-existing branch retinal venous obstruction has not been reported in published English literature.

Multicolour (MC) imaging is a non-invasive retinal imaging modality that simultaneously acquires three reflectance images using blue, green, and red confocal lasers, producing three separate and one composite image, visualising changes at different levels of the retina [5]. In Mac Tel type II, the blue reflectance (BR) image shows a characteristic parafoveal hyperreflectance [6]. We hereby present a case of concurrent MacTel with macular branch retinal venous obstruction and highlight the unique diagnostic features provided by BR and MC imaging.

## Case description

**Figure 1:**
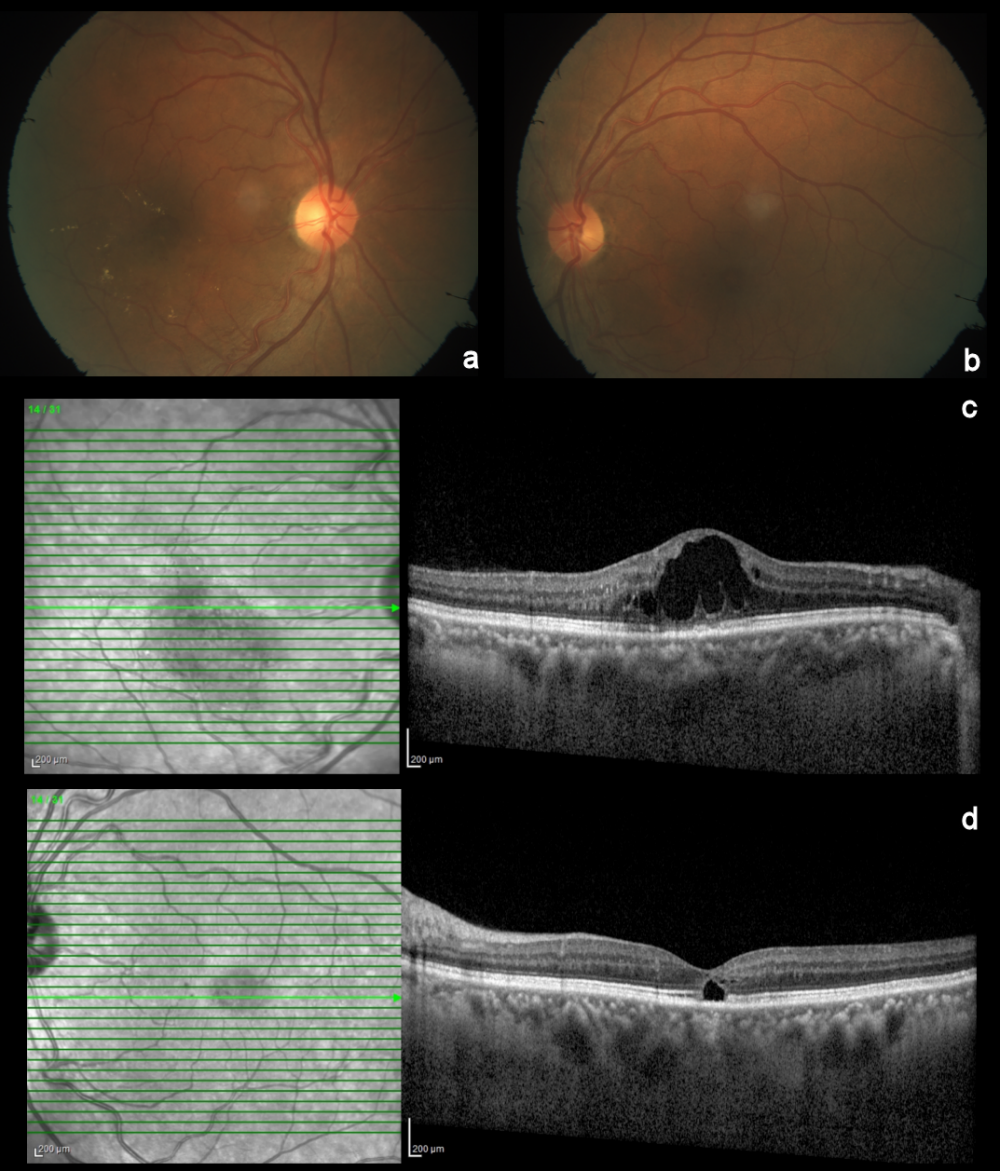
Color fundus photo and spectral domain optical coherence tomography of the right and the left eye. Colour fundus photo showing macular edema with hard exudates in the right eye (a), microaneurysms, RPE changes and a dull foveal reflex in the left eye (b). Horizontal spectral domain optical coherence tomography scan through the fovea revealed cystic changes with thickening of the fovea, hyperreflective dots corresponding to the hard exudates, and attenuation of the ellipsoid zone in the central and temporal parafoveal area of the right eye (c). In the left eye, spectral domain optical coherence tomography showed a central hyporeflective space in the outer retina with loss of the ellipsoid zone (d).

A 60-year old man reported with diminution of vision in the right eye since three months. He had a history of diabetes mellitus and hypertension since 3 years, both well controlled with oral medications. The best corrected visual acuity was 20/80 (+2.5 DS –0.75 DC 80°) in the right eye and 20/20 (+2.25 DS –0.75 DC 75°) in the left eye. The anterior segment was unremarkable in both eyes. Fundoscopy of the right eye showed edema with hard exudates over the macula (Figure 1a (Fig. 1)). The macula of the left eye showed retinal pigment epithelial changes (Figure 1b (Fig. 1)). The peripheral retina did not show any diabetic or hypertensive changes. Spectral domain optical coherence tomography (Heidelberg Engineering Germany) of the right eye revealed cystic changes with thickening of the fovea, hyperreflective dots corresponding to the hard exudates, and attenuation of the ellipsoid zone in the central and temporal parafoveal area (Figure 1c (Fig. 1)). In the left eye, spectral domain optical coherence tomography showed a central hyporeflective space in the outer retina with loss of the ellipsoid zone (Figure 1d (Fig. 1)). Central foveal thickness was 519 µm in the right and 203 µm in the left eye.

**Figure 2:**
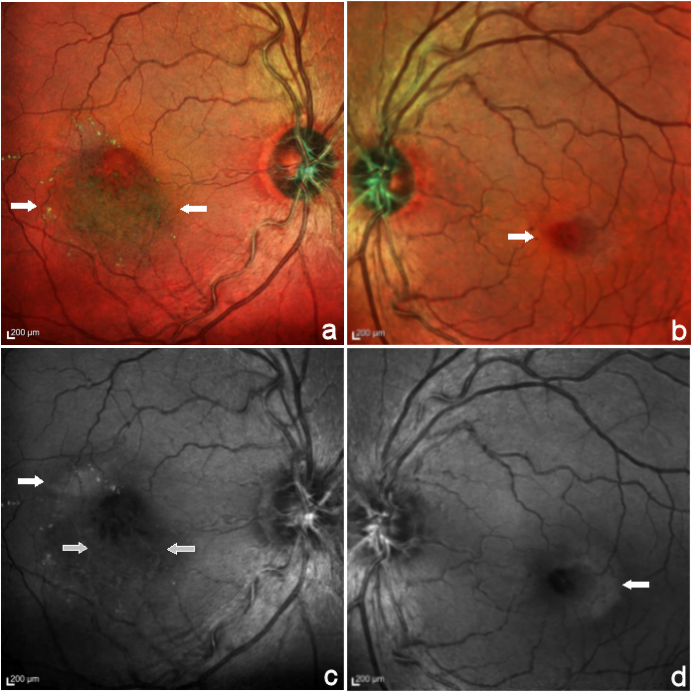
Multicolour composite images (MC) and blue reflectance images. The MC image showed greenish hue inferior to the fovea (between arrows) and yellow dots corresponding to the hard exudates (a). In the left eye MC composite image, the microaneurysms were clearly seen as dark red dots (arrow) (b). In the blue reflectance image, increased parafoveal reflectance was seen in both eyes (closed arrows) (c,d). Additionally, in the right eye it showed a clear distinction between hyperreflective vessels in the inferior parafovea and the hyporeflective appearance of other vessels in the blue reflectance image image (open arrows).

**Figure 3:**
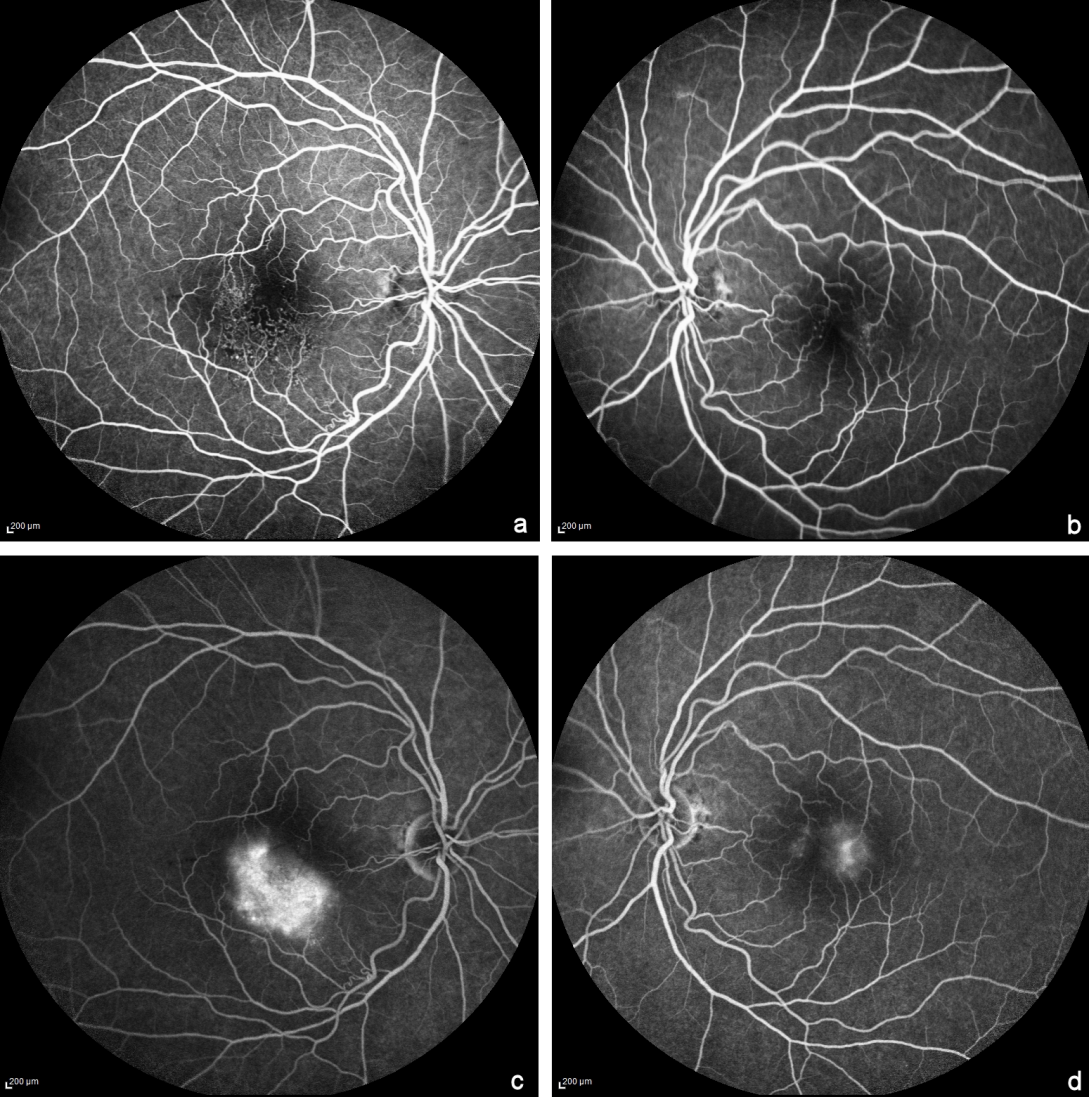
Fundus Fluorescein angiography early (a,b) and late phase (c,d). In the right eye, telangiectatic vessels in the temporal and inferior parafovea with few microaneurysms are seen in the early phase (a). In the left eye, microaneurysms in the parafoveal region are seen in the early phase (b). Late phase images show diffuse hyperfluorescence in the right eye (c) and mild staining of the temporal parafovea in the left eye (d).

MC imaging (Heidelberg Engineering, Heidelberg, Germany) of the right eye showed a greenish hue inferior to the fovea corresponding to foveal thickening on spectral domain optical coherence tomography and yellow dots corresponding to the hard exudates (Figure 2a (Fig. 2)). In the left eye, MC imaging showed the microaneurysms (Figure 2b (Fig. 2)). In the blue reflectance images, an increased parafoveal reflectance was seen in both eyes (Figure 2c,d (Fig. 2)). Additionally, in the right eye it showed a clear distinction between hyperreflective vessels in the inferior parafovea and the hyporeflective appearance of normal vessels in the blue reflectance images. Early phase fundus fluorescein angiography in the right eye showed telangiectatic vessels in the temporal and inferior parafovea with few microaneurysms and diffuse late phase hyperfluorescence that corresponded partly to the area of foveal thickening on optical coherence tomography and partly to the hyperreflective area on blue reflectance (Figure 3a,c (Fig. 3)). In the left eye, early phase fundus fluorescein angiography showed micoaneurysms in the parafoveal region and mild staining of the temporal parafovea in the late phase (Figure 3b,d (Fig. 3)). 

**Figure 4:**
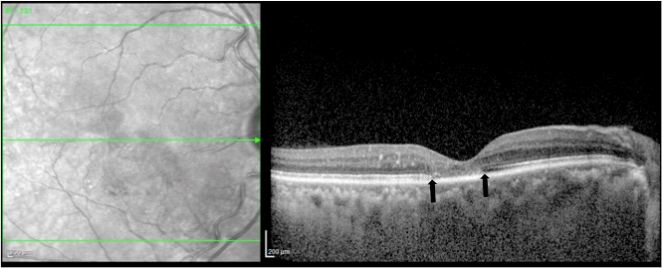
Horizontal spectral domain optical coherence tomography image through the fovea of the right eye one month after Ranibizumab therapy shows reduction of the central foveal thickness and complete resolution of the cystoid changes. Few hyperreflective dots corresponding to hard exudates are still seen and central loss of ellipsoid zone is seen centrally (between open arrows).

The right eye was treated with intravitreal Ranibizumab (0.5 mg in 0.05 ml). Four weeks post injection, macular thickness reduced to 144 µm and cystic changes disappeared on spectral domain optical coherence tomography. In addition, attenuation of the ellipsoid zone was seen (Figure 4 (Fig. 4)). Vision remained stable.

## Discussion

MacTel type 2 is a neurodegenerative disease with loss of foveal pigments and degeneration of Muller cells with secondary vascular changes like dilated telangiectatic vessels [2]. In contrast to patients with cystoid macula edema in diabetic retinopathy or branch retinal venous obstruction, patients with cystic spaces Mac Tel type 2 do not benefit from treatment with anti-vascular endothelial growth factors [7], [8]. Thus, distinguishing these different entities is important for treatment options. Furthermore, while treating cystoid macular edema in branch retinal venous obstruction, a presence of concurrent MacTel has implications for the visual prognosis as the macular edema might resolve but the visual loss due to MacTel will not improve.

Multicolor scanning laser imaging is an innovative non-invasive imaging technology. It provides structural information by using three individual laser wavelengths: blue (488 nm), green (515 nm), and infrared (820 nm) visualising changes at different levels of the retina [5]. The MC composite image uses false colors, which further helps in image interpretation. In MacTel, the blue reflectance image shows a characteristic parafoveal hyperreflectance due to the loss of foveal pigmentation early in the disease, making it a good tool for early detection [6]. Normal retinal vessels appear hyporeflective on multicolour images but pathological vessels can show hyperreflectivity [5], [9]. 

In the current report, parafoveal hyperreflectance on the blue reflectance image was seen in both eyes, characteristic of MacTel. Also, the blue reflectance imaging in the right eye in this case showed hyperreflective macular vessels that corresponded with the involved venules of the macular branch retinal venous obstruction seen on FFA. 

MacTel is diagnosed by characteristic features like parafoveal greying, crystalline depositions, and blunt venules which were masked by the exudates and retinal swelling in this case. The typical optical coherence tomography features were masked by large cystic spaces and increased retinal thickening, creating a diagnostic challenge. MC imaging provided vital diagnostic clues; increased parafoveal hyperreflectance and hyperreflective vessels to diagnose the two concurrent conditions in this case.

## Conclusion

The presence of multiple disorders can pose a diagnostic dilemma. In this report, we demonstrate the usefulness of MC imaging and in particular blue reflectance imaging as a non-invasive tool to identify concurrent MacTel and branch retinal venous obstruction. It also helps in guiding treatment decisions and counselling the patient about the disease prognosis. To the best of our knowledge, co-existence of MacTel and branch retinal venous obstruction has not been reported in published literature before.

## Notes

### Competing interests

The authors declare that they have no competing interests.

### Acknowledgements

The authors thank Moupiya Das and Marina Parveen (ophthalmic photographers).
